# Near-peers effectively teach clinical documentation skills to early medical students

**DOI:** 10.1186/s12909-022-03790-0

**Published:** 2022-10-08

**Authors:** Anita Vijay Kusnoor, Rajeev Balchandani, Malford Tyson Pillow, Stephanie Sherman, Nadia Ismail

**Affiliations:** Baylor College of Medicine, Houston, TX USA

**Keywords:** Near-peer, Clinical documentation, Resident as teacher

## Abstract

**Background:**

Composing the History of Present Illness (HPI), a key component of medical communication, requires critical thinking. Small group learning strategies have demonstrated superior effectiveness at developing critical thinking skills. Finding sufficient faculty facilitators for small groups remains a major gap in implementing these sessions. We hypothesized that “near-peer” teachers could effectively teach HPI documentation skills and fill the gap of small group facilitators. Here, we present a head-to-head comparison of near-peer and faculty teaching outcomes.

**Methods:**

Second-year medical students in a single institution participated in an HPI Workshop as a clinical skills course requirement. Students were randomly assigned a near-peer or faculty facilitator for the workshop. We compared mean facilitator evaluation scores and performance assessments of students assigned to either type of facilitator.

**Results:**

Three hundred sixty-five students, 29 residents (near-peers) and 16 faculty participated. On post-session evaluations (5-point Likert scale), students ranked near-peer facilitators higher than faculty facilitators on encouraging participation and achieving the goals of the session (residents 4.9, faculty 4.8), demonstrating small, statistically significant differences between groups. Mean scores on written assessments after the workshop did not differ between the groups (29.3/30 for a written H&P and 9/10 for an HPI exam question).

**Conclusions:**

Near-peer facilitators were as effective as faculty facilitators for the HPI Workshop. Utilizing near-peers to teach HPI documentation skills provided teaching experiences for residents and increased the pool of available facilitators.

## Background

Documenting the History of Present Illness (HPI), a critical skill for medical students, is an entrustable professional activity for entering clerkships [1] and residency [2]. Early clinical students often struggle with writing the HPI. We chose to use small groups to teach this critical thinking skill because small groups allow students to reflect and share individual experiences [3]. However, small group learning requires many facilitators, especially when the class size is large. We proposed utilizing “near-peers,” defined as “students who are more advanced, by at least one year distance, in the same curriculum” [4] to supplement the faculty facilitators. We hypothesized that near-peer teachers, such as residents, could suitably teach HPI documentation skills.

With regards to attainment of knowledge and skills, peer-teaching and faculty teaching have been shown to be equivalent [5]. Cognitive congruence theory and social congruence theory may explain the benefits of peer- and near-peer teaching. Cognitive congruence theory posits that near peers’ enhanced understanding of students’ cognitive problems makes them better able explain content in a manner that students can grasp. Social congruence theory suggests that learners may feel more comfortable revealing knowledge gaps or expressing uncertainty with near-peers than with faculty. Near-peers also serve as role models, provide reassurance, and allay some anxieties [4].

Near-peer teaching also benefits the teachers, who can practice teaching [5, 6], learn the subject better, and strengthen key clinical and professional skills [4, 7]. According to role theory, people in the role of teacher will assume the role of teacher, thereby gaining confidence [4].

Clinical documentation skills have become increasingly critical for medical students in the era of electronic charting. A recent study showed that near-peer feedback on clinical documentation improved students’ performance on the written note component of an Observed Structured Clinical Exam (OSCE) [8], but it lacked a comparison with feedback from faculty educators. We implemented a documentation skills workshop, the HPI Workshop, wherein students were randomly assigned to faculty- or resident- (near-peer) facilitated small groups. We compared students’ ratings of resident and faculty facilitators and compared student performance on formative and summative assessments.

## Methods

All second-year students participated in the HPI Workshop as a requirement for Patient, Physician and Society 3, a longitudinal clinical skills course. We recruited resident facilitators from the Academy of Resident Educators, a group of residents interested in education. The chief resident identified additional available residents. We recruited faculty facilitators from the course’s preceptors.

All facilitators received a facilitator guide before the workshop. Resident facilitators participated in a one-hour Teaching in Small Groups Workshop, during which a subject matter expert utilized and taught principles of small group teaching. They made connections between small group teaching strategies and activities planned for the HPI Workshop, which immediately followed.

In the workshop, students reviewed and corrected three sample HPI’s. They also critiqued each other’s documentation on an HPI from a preceptor session in the course. Subsequently, students and residents completed a post-session evaluation.

After the HPI Workshop, students submitted a written History and Physical (H&P) on a patient seen during a preceptor session. A cadre of 42 graders scored these H&Ps on a 30-point rubric in which the HPI was worth 7 points. For one station during the end-of-course summative OSCE, students wrote an HPI, which was graded on a 10-point scale by a single grader using a rubric.

We sought to determine whether students’ ratings of faculty and resident facilitators differed. We utilized Student’s t-test for continuous data to compare resident and faculty facilitator performance and student performance on the written H&P and the OSCE. We performed descriptive statistics on the residents’ post-session evaluation. All analyses were performed using Graphpad Prism Version 5. The Baylor College of Medicine Institutional Review Board determined this activity to be non-human subjects research.

## Results

Over two academic years (2018–2019 and 2019–2020), 365 students, 29 residents and 16 faculty participated. The response rate for the student post-session evaluation was 98%. Ninety-five percent of students agreed “The course should have this workshop again.” Responses did not differ by facilitator type.

The post-session facilitator evaluation asked students to rate their facilitator on a 5-point scale in which 5 meant “strongly agree” and 1 meant “strongly disagree.” Students ranked resident facilitators significantly higher than faculty facilitators on encouraging participation, making eye contact, addressing students by name and achieving the goals of the session (Table 1). Despite the statistical significance of these differences, the mean ratings were very close.

**Table 1:** Mean student ratings of faculty and resident facilitators

	**Residents** *N* = 209 (mean ± SEM)	**Faculty** *N* = 147 (mean ± SEM)	**P**
Facilitator encouraged participation	4.91 ± 0.02	4.82 ± 0.04	0.04
Facilitator made eye contact	4.88 ± 0.02	4.79 ± 0.04	0.05
Facilitator addressed students by name	4.84 ± 0.03	4.58 ± 0.08	0.001
The goals of the session were achieved	4.91 ± 0.02	4.83 ± 0.04	0.05

Response rate on the resident post-session evaluation was 66%. All residents agreed that the Teaching in Small Groups Workshop prepared them to facilitate and that residents should participate in this activity again.

Mean scores on the written H&P were 29.3/30 (SD 1.5) for students with faculty facilitators and 29.3/30 (SD 2.3) for students with resident facilitators. On the OSCE HPI question, mean scores were 9/10 (SD 0.9) for students in both groups.

## Discussion

In the current study, we found in a head-to-head comparison, that near-peers were equally effective to faculty educators in teaching clinical documentation skills. Students’ ratings of faculty and resident educators were similar, and students’ performance on formative and summative assessments was the same, regardless of whether they had a resident or faculty facilitator for the workshop. Our findings build upon those of numerous investigators, demonstrating that near-peers can effectively teach important clinical skills [5, 8]. Additionally, resident facilitators found the experience valuable, supporting prior evidence of benefit to near-peer teachers [7].

A variety of factors contributed to the success of near-peer facilitators in our HPI Workshop. Critically, all resident facilitators participated in the Teaching in Small Groups Workshop. In this workshop, faculty educators defined small group teaching, discussed its use in medical education, discussed the stages of group dynamics, and discussed the roles of participants and leaders in small groups. The faculty educators demonstrated techniques for engaging learners in small group settings and allowed residents to practice these techniques. Residents also reviewed the facilitator guide for the HPI Workshop in detail. The facilitator guide included the learning objectives, structure of the session, and a timeline for each activity in the workshop. The faculty educators answered the residents’ questions about the facilitator guide and shared details of their prior experiences facilitating the HPI Workshop. The success of faculty facilitators for the HPI Workshop, who did not attend the training session, may have been due to prior education or experience in small group teaching. Importantly, all facilitators were volunteers. Mandating participation may have yielded different outcomes. Other studies of near-peer educators also relied upon volunteers [5].

Our study had a strong design and included a large group of students, thereby improving power for comparisons. However, it was limited to a single institution. Though we were able to measure student performance on a written H&P and on an HPI on a summative OSCE, factors other than this workshop may have contributed to the students’ performance on these assessments. The graders for the H&P utilized a rubric, but we did not measure interrater reliability. We lacked objective measures of the outcome of resident training. For future development of this project, we may consider faculty or peer observation and feedback on resident teaching.

## Conclusions

Our Teaching in Small Groups Workshop prepared residents to facilitate small groups of medical students. Utilizing near-peers to teach HPI documentation skills provided teaching experiences with learner feedback to the residents, increased the pool of available facilitators for the workshop, and provided a high-quality educational experience for the students. Our findings should motivate programs to empower residents to do more teaching, providing benefits to the residents, the students, and the institution.

## Data Availability

The datasets used and/or analyzed during the current study are available from the corresponding author on reasonable request.

## Abbreviations

HPI: History of Present Illness
OSCE: Observed Structured Clinical Exam
